# Delivery of multipotent adult progenitor cells via a functionalized plasma polymerized surface accelerates healing of murine diabetic wounds

**DOI:** 10.3389/fbioe.2023.1213021

**Published:** 2023-08-22

**Authors:** S. J. Mills, G. T. Kirby, B. R. Hofma, L. E. Smith, P. Statham, B. Vaes, A. E. Ting, R. Short, A. J. Cowin

**Affiliations:** ^1^ Future Industries Institute, University of South Australia, Mawson Lakes, SA, Australia; ^2^ Cooperative Research Centre for Cell Therapy Manufacturing, Adelaide, SA, Australia; ^3^ ReGenesys BV, Bio-Incubator Leuven, Leuven, Belgium; ^4^ Athersys Inc., Cleveland, OH, United States; ^5^ Material Science Institute, Lancaster University, Lancaster, United Kingdom

**Keywords:** MAPC, Inflammation, wounds, diabetes, biomaterials, plasma polymerization

## Abstract

**Introduction:** Stem cell therapies have been investigated as potential treatment modalities for chronic wounds however there has been limited success to date. Multipotent Adult Progenitor Cells (MAPCs©) have been identified as having potential as an allogenic stem cell product due to their high population doubling number and their characteristic dampening of T-cell proliferation. This helps to prevent autoimmunity and graft/cell rejection.

**Methods:** We have developed a dressing, consisting of medical grade silicone coated with a heptylamine plasma polymer, which supports the growth and transfer of MAPCs to skin. To determine if the dressing can deliver functional stem cells into diabetic wounds, they were loaded with MAPCs and then placed over excisional wounds in both normal and diabetic mice.

**Results and discussion:** Accelerated healing was observed in both the normal and diabetic wounds with wound gape being significantly smaller at day 3 when compared to controls. Wound analysis showed that treatment with the MAPC dressings dampened the inflammatory response with reduced numbers of neutrophils and macrophages observed. Additionally, an increase in pro-angiogenic VEGF and CD31 positive endothelial cells was observed indicating improved new blood vessel formation. The MAPC dressings had no effect on fibrosis with collagen I and III being equally affected in both control and treated wounds. Overall, the functionalized MAPC dressings improve healing responses particularly in diabetic mice with impaired healing responses and therefore, show potential for development as an advanced therapeutic approach for the treatment of chronic diabetic wounds.

## 1 Introduction

Diabetes is one of the main causes of chronic wounds and the burden of this disease is rapidly increasing (Australia, 2021). There are thought to be around 422 million diabetic people and one in two adults undiagnosed with diabetes worldwide (Australia, 2021; Organisation, 2021). Approximately 15% of diabetic patients will go on to form diabetic foot ulcers and complications from these wounds lead to an occurrence of a major amputation every 30 s (Johannesson et al., 2009) with follow up mortality rates as high as 80%, over the next 5 years (Reiber et al., 2001). Clearly, there is an unmet demand for effective treatments, which improve healing of chronic wounds in diabetic patients.

The use of stem cells as a treatment for a variety of wound types has been around for some time. Mesenchymal Stem Cells (MSCs) are a cell type that have been widely used for this purpose due to their multipotent characteristics enabling them to differentiate into many different cell types including, chondrocytes, adipocytes, tenocytes, and myocytes (Kirby et al., 2015). The majority of studies show that treatment of a wound with MSCs accelerates the rate of wound closure in both normal and diabetic mice (Badiavas and Falanga, 2003; Ichioka et al., 2005; Mansilla et al., 2005; Falanga et al., 2007; Dash et al., 2009; Stoff et al., 2009; Mills et al., 2013; Gao et al., 2014; Huang et al., 2015; Ahangar et al., 2020a; Thomas et al., 2021). This improvement in healing is achieved via increases in vascularization, cellularity and elastin production as well as reduction in scar formation. Isolated human MSCs have been delivered to wounds using a variety of different methods. Badiavas and Falanga (2003) isolated the patients MSCs, via a bone marrow aspirate of the iliac crest. These cells were then administered directly onto a debrided wound site and were also injected intradermally into the wound margins. A remainder of the cells were also cultured, and three additional treatments were administered. This treatment was applied to three patients, all of which had chronic cutaneous ulcerations. The MSC treatment resulted in wound closure, with increased vascularization, in all three patients with no reoccurrence of the wounds after following up 1-2 years later (Badiavas and Falanga, 2003). Falanga et al. (2007) refined their treatment in this later study by isolating the mononuclear cells from the iliac crest, and then culturing them in MSC media. These cells were then characterised before being incorporated into a fibrin spray containing 5 mg/mL fibrinogen and 25 U/mL thrombin. This cell suspension was then sprayed topically onto the surface of acute and chronic human wounds. The acute wounds were shown to heal with some of the MSCs incorporated into the newly formed tissue. The chronic wounds were shown to reduce in wound area with the MSCs again remaining in the wound site. MSCs isolated from mice and applied using the fibrin spray technique were also able to accelerate healing in diabetic mice (Falanga et al., 2007). Ichioka et al. (2005) investigated the use of a collagen matrix to delivery their bone marrow cells (Ichioka et al., 2005). The cells were isolated from the bone marrow of mice and then impregnated into the collagen matrix. The cells using in this study were not passaged or characterised and are more likely a mixed population of stromal cells rather than an enriched population of MSCs. The collagen matrix containing the cells was then placed in a wound created using a skin fold model of healing, which allowed the visualization of new blood vessel growth. Treatment with the bone marrow suspension showed increased vascularization of the murine wounds. This method of treatment was also used for the treatment of a human non-healing ulcer. After treatment there was an induction of granulation tissue formation that led to the eventual closing of the wound (Ichioka et al., 2005). Human MSCs have also been shown to have low immunogenicity and promote healing in incisional wounds in rabbits (Stoff et al., 2009). In this study, 3 cm incisional wounds were created on the dorsum of New Zealand White rabbits. The wounds were sutured and then fluorescently labelled human MSCs were intradermally injected around the wound site. After 7 days the sutures were removed, and the wounds left to heal. The wounds treated with hMSCs were shown to have increased tensile strength and reduced scarring (Stoff et al., 2009).

Other studies with human MSCs and stromal cells also showed that in addition to their beneficial effects on the wound healing process in skin they can also improve healing in other tissues such as the spinal cord and the myocardium (Mansilla et al., 2005; Huang et al., 2010). Their effects are wide ranging and have also shown to alter lipid metabolism during wound healing in diabetic wounds (Gao et al., 2014). However, despite the numerous studies being performed using MSCs, several differences in how the cells are characterized, where they are harvested from, their dosage and method of application has hindered their uptake or application (Kirby et al., 2015). Additionally, the term “MSC” in the literature has also referenced mesenchymal stromal cells, which unlike stem cells are a heterogenous population of different cell types, of which only a small population may be true mesenchymal stem cells. Whilst mesenchymal stem cells can help to repopulate lost cells and tissues the mesenchymal stromal cell population acts more by homing to the wound and secreting factors which are often immunomodulatory in nature (Viswanathan et al., 2019). This confusion and poor characterization has led to an increasing number of treatments with a high degree of variability, which has potentially hindered the approval of MSC treatments available for the treatment of chronic wounds (Moll et al., 2019; Moll et al., 2022).

Given the lack of progress with the development of MSC treatments, other types of stem cells have been investigated. Multipotent adult progenitor cells (MAPCs) are bone-marrow derived non-haematopoietic adherent cells that have been identified as a potential allogenic cell type for the treatment of various conditions (Boozer et al., 2009; Reading et al., 2013; Laing et al., 2020; Carty et al., 2021). They were first described in 2002, by Jiang et al., and were found to have similar characteristics to MSCs in that they could proliferate without senescence and differentiate into the three germ layers to create bone, cartilage, fat, muscle, tendon, endothelium, neurons, glia and hepatocytes (Jiang et al., 2002; Khan and Newsome, 2019).

One advantageous characteristic of MAPCs, not shared by MSCs, is that they can be expanded for over 70 population doublings, unlike MSCs which are limited to around 20–25 doublings. This makes MAPCs much more robust when expanding the population for *in vivo* treatments and for upscaling during manufacturing of a therapeutic (Roobrouck et al., 2011). MSCs and MAPCs have also been shown to be non-immunogenic as they lack the expression of MHC class II and have low expression of MHC class I molecules, as well as costimulatory markers (CD40, CD80, and CD86), and CD45 (Khan and Newsome, 2019). Interestingly, it has been observed that upon differentiation or exposure to IFNγ, MSCs increase their expression of MHC class I and class II molecules making them susceptible to lysis by natural killer cells (Le Blanc et al., 2003). MAPCs, however, upregulate MHC class I but not class II molecules and they even inhibit host IL-7-dependant effector T-cells proliferation, which may prolong their persistence (Tolar et al., 2006; Jacobs et al., 2013; Reading et al., 2015). This dampening of the immune response and inhibition of T-cell proliferation, brought about by MAPC expression of prostaglandin E2, suggests that potentially MAPCs make an ideal allogenic product and is why they are currently being tested in graft versus host disease, immunomodulation after liver transplant and for cardiac regeneration (Pelacho et al., 2007; Highfill et al., 2009; Jacobs et al., 2013; Soeder et al., 2015).

MAPC actions, *in vivo*, appear not to be limited to immunomodulatory properties but they also have beneficial effects on existing vasculature as well as promoting new vessel growth. This has been observed in a variety of models including islet cell transplantation were significantly increased VEGF expression and vascularization was observed. This resulted in an increased number of islet cells that survived engraftment (Cunha et al., 2017). This improvement in vascularization has also been shown in a model of limb ischaemia where treatment with MAPCs led to improved vascularization and blood flow with improved vascular and skeletal muscle cell growth repairing the ischaemic damage (Aranguren et al., 2008). MAPCs have also been shown, via proteomic analysis, to regulate a wide variety of proteins when stimulated with either IFNγ, LPS or (a tolerogenic CD74 ligand) RTL1000 (Burrows et al., 2013). The functions of these proteins included in processes such as extracellular matrix formation and regulation (MMPs and proteases), angiogenesis and immune regulation of cells such as neutrophils, macrophages and T-cells (Burrows et al., 2013). MAPCs not only possess many of the beneficial traits of their MSC counterparts, when used for the treatment of a wide variety of disorders, but they also have selective advantages over MSCs when being considered for an allogenic therapeutic. The increased number of cells cycles that they can be expanded for mean that MAPCs are more robust when generating the required number of cells for *in vivo* treatment. In addition, their dampening of the immune response means that they can persist within the body to prolong their beneficial effects on the immune response and vascularization process resulting in better treatment outcomes when compared to MSCs.

When developing stem cell products for the treatment of acute and chronic wounds the effects of the mode of delivery and its optimization is often not fully considered. A common method of delivering stem cells is with the cells in suspension (Basiouny et al., 2013), however this is often preceded by enzymatic digestion, which can be detrimental to the cells. Cells are also administered via injection but this technique has been shown to increase rates of apoptosis in the delivered cells (Amer et al., 2015). This has led to interest in producing stem cell therapies which avoid enzymatic digestion and deliver the cells as a sheet (Kirby et al., 2017). We have previously described the manufacture of a functionalized heptylamine surface on a silicone backing, using plasma polymerization, that can transfer MAPCs onto a dermal equivalent *in vitro* (Kirby et al., 2017). This dressing negates the need for any enzymatic digestion prior to use and the functional surface allows the cells to detach and migrate into the wound site (Kirby et al., 2017). The functional effects of MAPCs and this mode of delivery suggests that the functionalized dressings with adherent MAPCs may be beneficial for the treatment of diabetic wounds which have impaired healing.

Here, for the first time, we describe the delivery, and optimization of dosage, of MAPCs using a functionalized dressing for wound healing. Using preclinical wound models in normal and diabetic mice we have shown that MAPC dressings improve healing outcomes through a dampening of the immune response and an increase in angiogenesis. In addition, we also showed that when MAPCs are delivered to the entire surface of the wound via the dressing that healing is accelerated when compared to injecting MAPCs intradermally around the perimeter of the wound.

## Material and methods

### Chemical and reagents

Primary antibodies NIMP-R14 (sc-59338) and MRP-14 (sc-8114) were from Santa Cruz Biotechnology (United States), collagen I (600-401-103) and collagen III (600-401-105) were from Rockland Immunochemicals Inc. (United States), VEGF (MA1-16626) was from Thermo Fisher Scientific (MA, United States) and CD31 (ab28364) and HNA antibody (ab215396) was from Abcam (Cambridge, UK). All secondary antibodies were Alexa Fluors, goat anti-rat 660 (A21054), donkey anti-goat 594 (A11057) and goat anti-rabbit 488 (A11008) and IgG antibody (02-6502) were from Thermo Fisher Scientific (MA, United States). Streptozotocin (STZ; S0130) and heptylamine were sourced from Sigma-Aldrich (MO, United States). Silicone was obtained from Polymer Systems Technology Ltd. (High Wycombe, UK).

### Functionalized dressing manufacture

**FIGURE 1:**
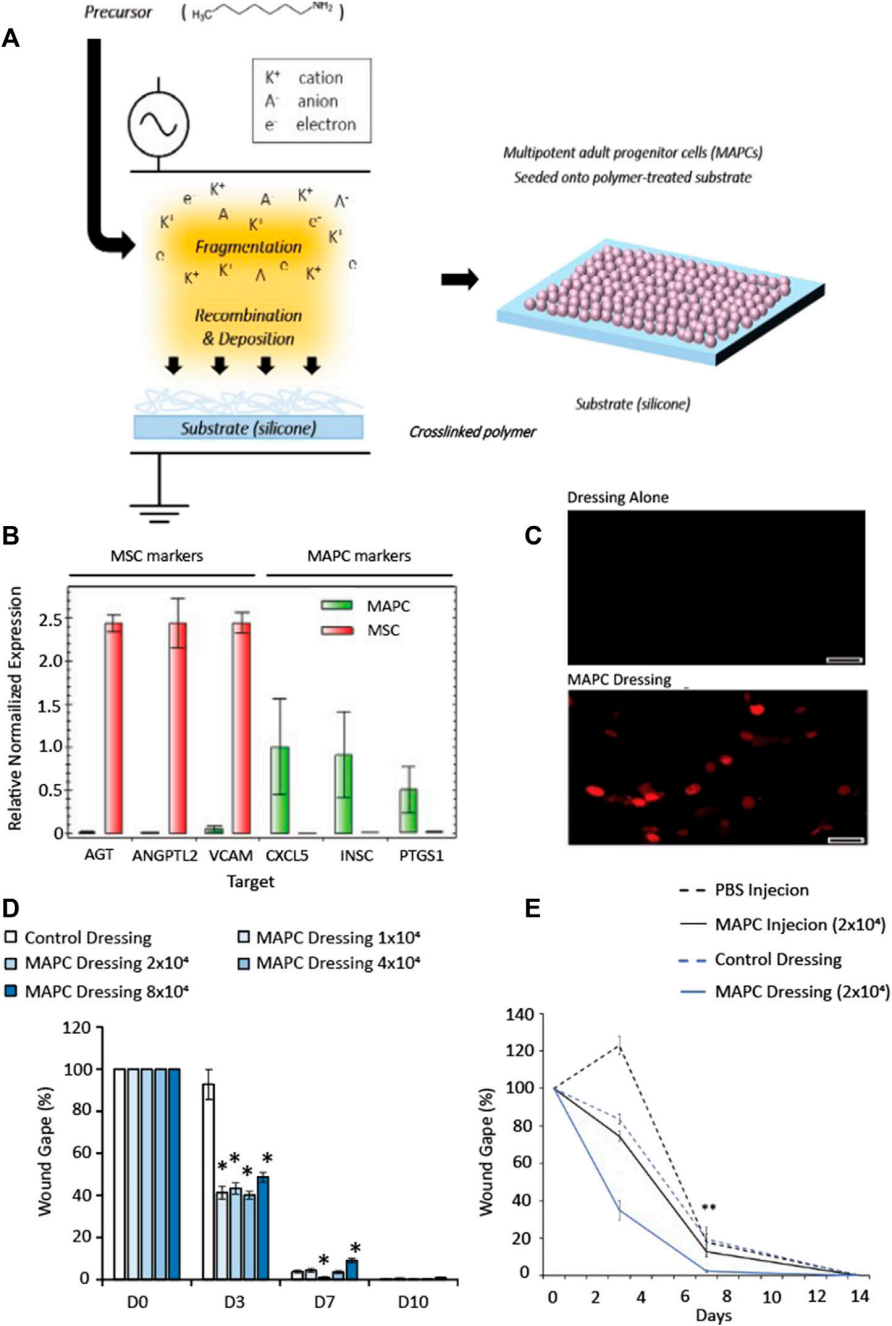
Schematic representation showing the fabrication steps for the cell-dressing **(A)**. A radio-frequency plasma polymer process is used to generate a chemically functional polymer onto the silicone dressing (A, left). Multipotent adult progenitor cells are then seeded onto the dressing and allowed to adhere for 24 h (A, right). **(B)** Expression of MAPC and MSC markers of donor matched MAPCs and MSCs to confirm the genotypes of the cells. **(C)** HNA staining of human MAPC cells in mouse wounds in MAPC treated and dressing alone at day 7 (scale bars = 50 µm). **(D)** Dose response of MAPCs in diabetic wounds. **(E)** Comparison of wound gape from diabetic mouse wounds injected at D0 with 2 × 10^4^ MAPC or treated with a dressing seeded with 2 × 10^4^ MAPC. Markers of significance are * = *p* ≤ 0.05, and ** = *p* ≤ 0.01. The data in **(D,E)** was compared using a one-way ANOVA with a Bonferroni correction.

Dressings were manufactured using a backing substrate of 0.3 mm-thick silicone, which were coated with heptylamine using plasma polymerization and a parallel plate reactor (Figure 1A). A radio frequency power of 13.56 MHz was used along with an initial pressure of 2 × 10^−2^ mbar. The deposition time was 20 min, and the precursor flow was maintained for a further 10 min. The method and physico-chemical analysis of the polymer produced is more fully described in Kirby et al. (2017) (Kirby et al., 2017).

### Cell culture

Human MAPCs were obtained from ReGenesys (Heverlee, Leuven, Belgium), prepared from the bone marrow of a healthy volunteer and cultured under previously described conditions (Boozer et al., 2009). Briefly. MAPCs were expanded on a primed and fibronectin-coated hollow-fibre cartridge of the Quantum cell expansion system (TerumoBCT, NJ, United States), harvested using trypsin/EDTA (Lonza, Basel, Switzerland), cryopreserved and stored in the vapour phase of liquid nitrogen until use. Cells were further expanded on fibronectin-coated plastic tissue culture flasks. Cell cultures were maintained under low oxygen tension in a humidified atmosphere of 5% CO_2_. Cells were cultured to sub-confluence in MAPC, low glucose DMEM, culture media (Thermo Fisher Scientific, MA, United States) supplemented with FBS (Atlas biologicals, CO, United States), MCDB (Sigma-Aldrich, MO, United States), platelet-derived growth factor (R&D Systems, MN, United States), epidermal growth factor (R&D Systems, MN, United States), dexamethasone (Sigma-Aldrich, MO, United States), penicillin/streptomycin (Invitrogen, CA, United States), 2-Phospho-L-ascorbic acid (Sigma-Aldrich, MO, United States), and linoleic acid–albumin (Sigma-Aldrich, MO, United States). Cells were imaged live using phase contrast microscopy and were split when they reach confluence using phosphate buffer saline (PBS, Lonza, Basel, Switzerland) and trypsin-EDTA (Lonza, Basel, Switzerland). Prior to use, cells were washed in PBS and seeded onto the functionalized dressings using a seeding ring, to keep the cells within a defined area. To achieve this a 10 mm seeding ring with an area of 0.8 cm^2^ was used and either 1 × 10^4^, 2 × 10^4^, 4 × 10^4^ or 8 × 10^4^ cells were seeded per dressing. After 24 h the seeding ring was removed, and the dressing was ready for use.

### mRNA isolation and analysis

Cell expression of MAPC and MSC markers were assessed via mRNA isolation and qRT-PCR as previously described (Kirby et al., 2017). In brief, donor matched MSCs and MAPCs were cultured, and RNA extracted using an AllPrep Protein/RNA Isolation Kit according to the manufacturers protocol (QIAGEN, Germany). The RNA was quantified using a NanoDrop Lite Spectrophotometer (Thermo Fisher Scientific, MA, United States) and the expression of the following markers was assessed via qRT-PCR: AGT, VCAM1, ANGPTL2, VCAM, CXCL4, INSC, and PTGS1. Expression was normalised to the housekeeping gene ATP5B following iScript cDNA synthesis (BioRad, CA, United States) and qPCR on the BioRad CFX Connect using the SsoAdvanced SYBR Green Universal Supermix synthesis (BioRad, CA, United States).

### Animal studies

All animal studies were carried out with approval from the Women’s and Children’s Health Network Animal Ethics Committee (secondary healing model—AE971/4/2018 and diabetic healing model—AE984/6/2017) and carried out in accordance with the Australian code of practice for the care and use of animals. Mice were all eight-to ten-week-old females (BALB/C) sourced from the Animal Resources Centre (Perth, WA, Australia). Mice were acclimatized for 7 days upon arrival and were kept in a 12-h light/dark cycle, in a temperature and humidity-controlled environment. Only female mice were used as there are significant inherent difference in the wound healing mechanism when compared to male mice. It has been reported in the literature that oestrogen can accelerate healing via a dampening of the inflammatory response whilst androgen have the opposing effect (Ashcroft and Mills, 2002; Ashcroft et al., 2003). Studies in male mice will be conducted if the MAPC treatment is successful.

### Diabetes induction

10–12-week-old mice were fasted 4 h prior to streptozotocin (STZ) being injected interperitoneally at 50 mg/kg, in citrate buffer pH 4.5 (100 ul), for 5 consecutive days. The mice were then group housed and mushy food was supplied until healthy weights were maintained. Blood glucose levels were tested daily and 1-2 U of insulin was administered as required to maintain the blood glucose levels within the diabetic range of 15–25 mmol/L. Following maintenance for 6 weeks post-STZ injection, only mice within the diabetic range for the entirety of weeks 4–6 were included in the study. Overall, 70% of the mice in this study became diabetic using this method and these were included in the diabetic groups to give *N* = 7. The other 30% of the non-diabetic mice were used for other studies.

### Surgical procedures

Groups of healthy and diabetic mice were anaesthetized with inhaled isoflurane. Mice were initially placed in an induction box supplied with at 2 L/min of oxygen and 3% isoflurane. Once under surgical depth anaesthesia the mice were transferred to a nose cone which was supplied with 0.2 L/min oxygen and 2% isoflurane. At this point the dorsum was shaved, depilated with Veet cream and cleaned with 70% ethanol. Two equidistant 6 mm full thickness excisional wounds were created, using a punch biopsy, on the dorsal surface, 1 cm from the base of the skull and 1 cm either side of the midline. Each wound was then covered with a circular 1 cm diameter dressing which was held in place using a 3 cm by 1 cm strip of Tegaderm. Mice were under anaesthetic for an average of 5 min.

### MAPC and control treatments

Treatments were either administered as intradermal injections or via the functionalized dressing. For the intradermal injection the treatment consisted of MAPCs in PBS or PBS alone, with a total volume of 100 uLs. 25 μLs of PBS or the cell suspension was injected intradermally 2 mm from the wound margin, at 4 sites equidistant around the wound circumference. The dressings were either the functionalized surface alone or the functionalized surface seeded with MAPCs. Both the wounds on the mice received the same treatment for all studies i.e., either control dressings or inject MAPCs or dressing seeded with MAPCs or PBS injection. The dressings were removed after 72 h, and the wounds left to heal by secondary intention i.e., no sutures or dressings were used after 72 h. *N* = 7 mice per group were used as calculated using a power calculation.

### Dose response study

An initial *in vivo* wound healing study was performed to identify the optimal dose of MAPCs that would result in the fastest rate of wound closure. The wounding was carried out as described in the surgical procedures section in diabetic mice and five groups were compared, a control functionalized dressing with no cells and dressing seeded with either 1 × 10^4^, 2 × 10^4^, 4 × 10^4^ or 8 × 10^4^ cells. Macroscopic wound gape measurements were then compared to determine the rate of healing.

### Comparison of MAPC delivery

In this study using diabetic mice, MAPCs were delivered to the wound either via intradermal injection or via the functionalized dressing. The controls used were wounds treated with PBS delivered via an intradermal injection or a functionalized dressing with no MAPCs. MAPCs in PBS were initially delivered to the wound surface but the cell suspension was not retained within the wound site due to a lack of viscosity as such it was decided to deliver the cells via intradermal injections.

### Acute and diabetic wound healing studies

These studies were carried out as specified in the surgical procedures section and the MAPC dose used was 2 × 10^4^ cells per wound. The two treatment groups were the functionalized dressing seeded with MAPCs or the functionalized dressing alone.

### Macroscopic assessment

All wounds were photographed daily, and macroscopic measurements of re-epithelialization, termed wound gape, were measured from these digital images.

### Microscopic assessment

Wounds were collected from groups of mice on days 3, 7 and 14 and bisected. Half the wounds were snap frozen in liquid nitrogen and used for molecular analysis and half the wounds were fixed for 24 h in 10% normal buffered formalin before being processed into paraffin and used for histological analysis. 5 µm wound sections cut and mounted onto coated glass slides. Slides were stained with haematoxylin and eosin for microscopic wound measurements including, wound width, wound area and percentage re-epithelialization.

### Immunohistochemical staining

5 µm sections on glass slides were placed into xylene before the sections were rehydrate using graded concentrations of ethanol. Antigen retrieval was carried out in 10 mM citrate buffer, pH 6.0, where the sections were heated to 90°C for 10 min. Once cooled the sections were incubated with an appropriate blocking serum for 30 min. Primary antibodies to NIMP-R14, MRP-14, VEGF, CD31, HNA, collagens I and III were diluted 1:100 using PBS and secondary antibodies were diluted 1:200 in PBS. Control staining used an appropriate IgG antibody, which was used in place of the primary antibody. Secondary antibodies were Alexa Fluor, mouse anti-rat 660, mouse anti-goat 594 and mouse anti-rabbit 488. All sections were counterstained with DAPI (4’, 6-diamidino-2-phenylindole) at a concentration of 1:5000. Images were captured on Olympus IX83 with a DP80 camera, at ×20 magnification. Analysis and quantification of cells per unit area (mm^2^), fluorescent intensity and wound measurements were performed using CellSens software (Olympus, Japan).

### Statistical analysis

Statistical significance was calculated using a two-tailed Student’s t-test when comparing two groups. A one-way ANOVA was performed, with a Bonferroni correction, for comparison of multiple groups. A *p*-value of less than 0.05 was considered significant.

## Results

### Expression of MSC and MAPC panel markers

To confirm the population of cells used were MAPCs, analysis of expression markers for donor matched MSCs and MAPCs was performed. Significantly more expression of MSC markers (AGT, ANGPTL2, and VCAM) were observed in the MSC cells compared to MAPCs, which had little measurable expression (Figure 1B). The expression of the MAPC markers (CXCL5, INSC and PTGS1) had greater expression in the MAPCs when compared to the MSCs, which had little or no expression of these markers (Figure 1B).

### Identification of the optimal dose of MAPCs delivered using the functionalized dressings to treat diabetic wounds

Wounded diabetic mice (*n* = 7) were treated with functionalized dressings seeded with: 1 × 10^4^, 2 × 10^4^, 4 × 10^4^, 8 × 10^4^ MAPCs or functionalized dressing (cell-free) alone. Macroscopic images were taken at day 0, day 3, day 7 and day 10 and wounds harvested at day 7 and day 10. To confirm delivery of human MAPCs into the murine wounds, human nuclear antigen (HNA) antibody staining was performed. The presence of human MAPCs was confirmed at day 7 with HNA positive cells observed within the wounds (Figure 1C). Macroscopic wound measurements showed that at day 3 all mice treated with MAPC dressings had smaller wounds when compared to those treated with cell-free control dressings with wound gape being 44.1%–52.7% smaller than the dressing alone depending on the dosage used. At day 7, only wounds treated with the 2 × 10^4^ MAPC dressing remained significantly smaller than wounds with dressing alone (*p* = 0.0103) (Figure 1D). In addition, wounds treated with the highest dose of MAPCs showed delayed healing compared to those treated with the cell-free dressings (*p* = 0.0009). Due to this data all other studies used a dosage of 2 × 10^4^ MAPCs as this was shown to be most effective at promoting wound repair.

### MAPCs delivered using functionalized dressings improved healing when compared to injected MAPCs

To determine if injection of MAPCs was as effective as delivery via a functionalized dressing, wounded diabetic mice (*n* = 7) were either treated at day 0 with 2 × 10^4^ MAPC injected intradermally at four sites around the wound margin or a functionalized dressings seeded with 2 × 10^4^ MAPCs. Two control groups of injected PBS or a dressing alone treatment were also included. At day 3, wounds treated with the MAPC dressings (34.9%) had smaller wounds when compared to wounds treated with MAPC injections (74.5%), PBS injections (122%) and dressing alone (83.6%), though this data was not significant. Wounds treated with the MAPC dressings (2.38%) were significantly smaller at day 7 (*p* = 0.003) when compared to wounds directly injected with equivalent numbers of MAPCs (12.76%) or the PBS (17.8%) and dressing alone (19.4%) groups (Figure 1E).

Figure 1E showed a trend to suggest that the wounds treated with injected MAPCs, or the dressing alone were healing at a faster rate than the PBS injected wounds, at day 3. This, however, was not significant when the data was compared by one-way ANOVA. It has been reported in the literature that plasma polymerized surfaces can promote the proliferation of human keratinocytes, which may explain the perceived reduction in wound area of the wounds treated with the dressing alone, when compared to the PBS group at day 3 (Haddow et al., 2003).

Interestingly, the wounds treated with the MAPC dressing had a wound gape of 34.9% and 2.4%, at day 3 and 7 respectively, whereas the MAPC injected wounds had a wound gape of 74.5% and 12.7%. These observed differences may be because the dressing administration delivered the MAPCs to the entire wound surface whereas the intradermally injected MAPCs were only delivered into the wound margins and had to migrate into the wound site. There are also reports in the literature that show up to 80% of a dose delivered via injection can be non-viable depending on the needle size and flow rate used (Amer et al., 2015). This may also account for the reduction in efficacy seen with the injected MAPCs. As a comparatively low dose of MAPCs was administered to each wound (2 × 10^4^ cells per wound) any loss via injection could significantly impact the effect of the MAPC treatment. In addition to this, it would result in differing doses being delivered to the wound site when comparing the intradermal injection and the dressing delivery system. As such the intradermal injection was not considered a reliable control and only the dressing alone control was used for the remainder of the study.

### Delivery of MAPCs improves macroscopic healing in both normal and diabetic wounds

**FIGURE 2:**
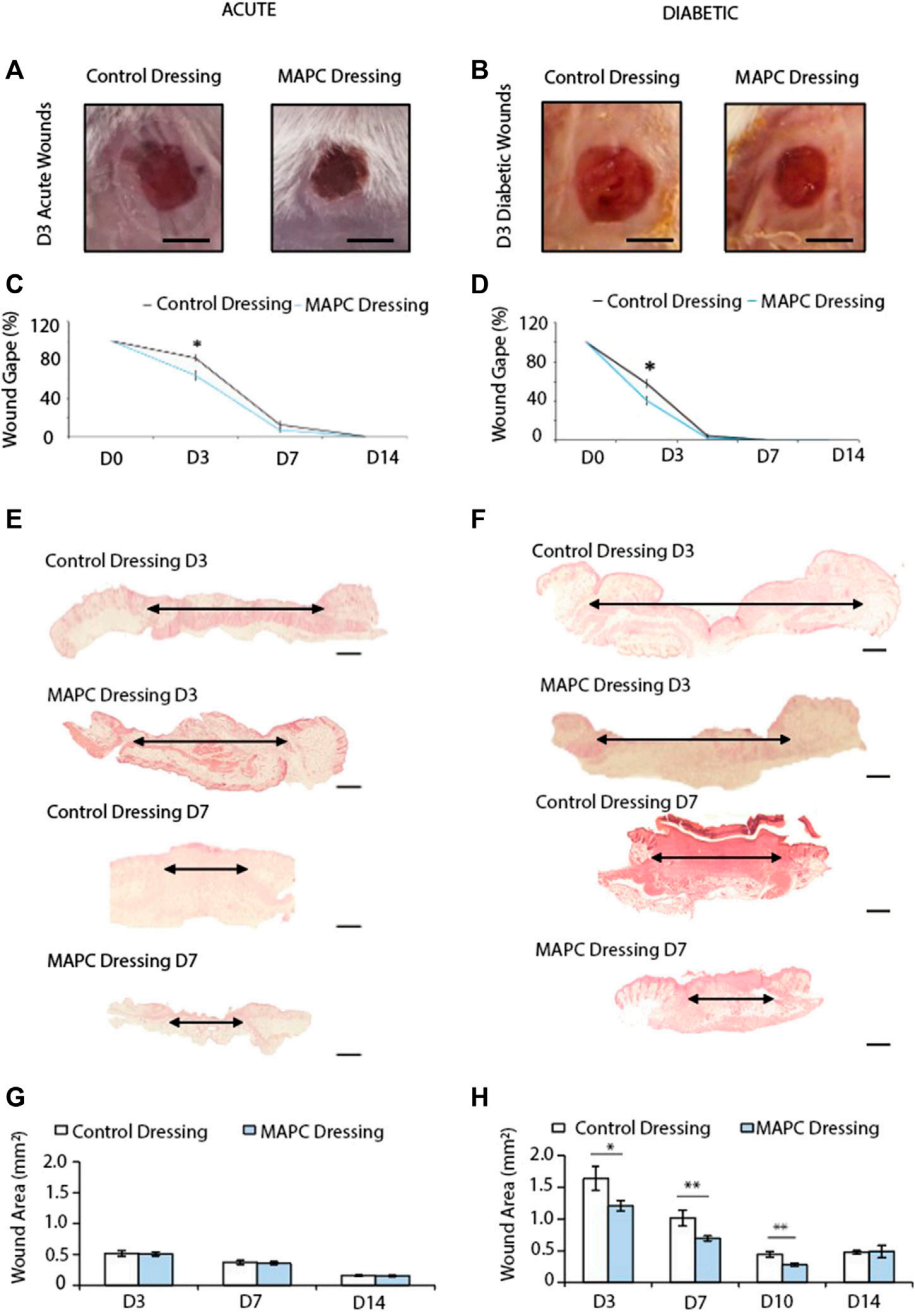
Representative images of the macroscopic appearance of the day 3 normal **(A)** and diabetic **(B)** wounds treated with MAPC dressings and control dressings (Scale bars = 2 mm) and the respective macroscopic wound gape measurements **(C,D)**. Histological H&E images of day 3 and day 7 normal **(E)** and diabetic **(F)** wounds treated with MAPC dressings or control dressings (scale bars are 20 µm) and their respective microscopic wound area measurements **(G,H)**. Markers of significance are * = *p* ≤ 0.05, and ** = *p* ≤ 0.01. Significance was determined using a two tailed student t test.

In both normal and diabetic mice, the delivery of MAPCs by the functionalized dressings significantly accelerated macroscopic wound closure at 3 days post-wounding (Figures 2A–D). At day 3, excisional wounds in normal mice treated with MAPC dressings had reduced by 26.9% (Figure 2C). This was significantly smaller (*p* = 0.021) than the 17.9% reduction observed in the control wounds (Figure 2C). This improvement was also seen in diabetic mice treated with the MAPC dressing with wound gape being significantly reduced by 59.8% compared to wounds treated with control dressings which were reduced by 36.3% (*p* = 0.001) (Figure 2D). There were no significant differences seen at day 7 in either the normal or diabetic wounds.

### MAPC dressings decrease wound area in diabetic but not normal wounds

When wound area in normal mice was assessed microscopically, no differences were observed at any of the time points between wounds treated with MAPC dressings or control dressings (Figures 2E, G). Diabetic wounds have impaired healing, and the wounds were significantly larger than those in normal healthy mice. When treated with MAPC dressings, a decrease in wound area was observed, in the diabetic mice, of 26% at day 3 (0.43 mm, *p* = 0.04), 31.5% reduction at day 7 (0.32 mm, *p* = 0.02) and 35.9% at day 10 (0.15 mm, *p* = 0.002) when compared to control dressings (Figures 2F, H).

### Reduced neutrophil influx in normal and diabetic wounds treated with MAPC dressings

**FIGURE 3:**
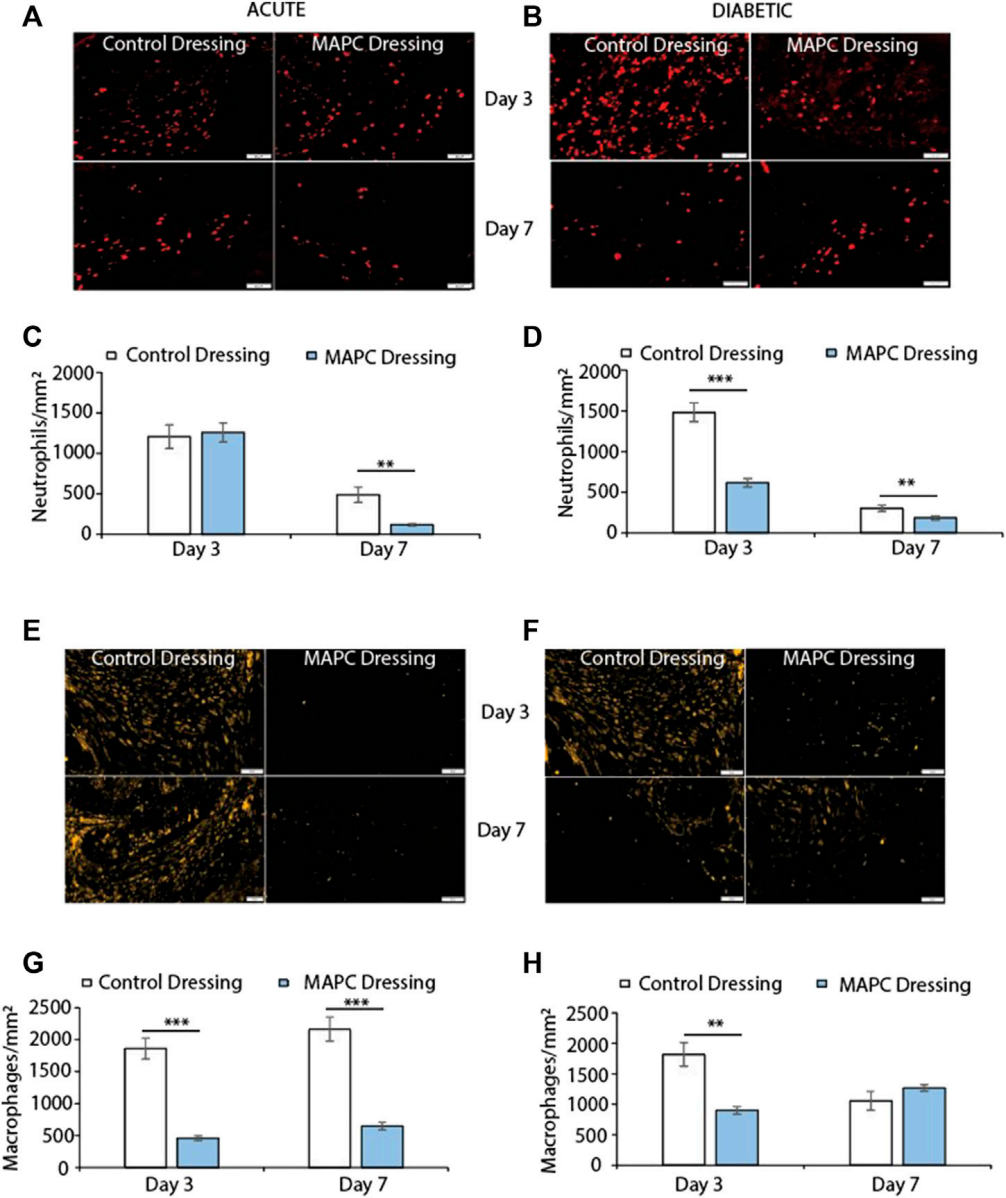
Representative images showing NIMP-R14 positive neutrophils at day 3 and day 7 in normal **(A)** and diabetic **(B)** wounds treated with MAPCs dressings or control dressings and their respective positive cell staining counts per mm^2^ **(C,D)**. Representative images of the immunohistochemical staining of MRP-14 positive macrophages in day 3 and day 7 normal **(E)** and diabetic wounds **(F)** and their cell counts per mm^2^ **(G,H)**. Markers of significance are ** = *p* ≤ 0.01 and *** = *p* ≤ 0.001 and scale bars are 50 µm. Significance was determined using a two tailed student t test.

Neutrophils are among the first immune cells to immigrate into the wound site and whilst there were no significant differences observed in the day 3 excisional wounds in normal mice there was a significant reduction (*p* = 0.002) in neutrophilic persistence in the MAPC dressing treated wounds when compared to control wounds at day 7 (Figures 3A, C). In contrast, a significant reduction in neutrophilic influx at day 3 (*p* = 0.0008) was observed in MAPC dressing treated diabetic wounds and persistence at day 7 (*p* = 0.006) was again observed compared to control dressings (Figures 3B, D).

### MAPC dressings modulate macrophage influx into normal and diabetic wounds

Excessive macrophage recruitment to the wound site is often linked with a delayed wound healing phenotype (Kirby et al., 2015). In the normal mice wounds, treatment with MAPC dressings significantly reduced the numbers of macrophages at both day 3 (*p* = 0.0005) and day 7 (*p* = 0.001) when compared to control wounds (Figures 3E, G). This reduction in macrophages was also seen at day 3 in the diabetic wounds (*p* = 0.003) but was not maintained at day 7 (Figures 3F, H).

### Treatment with MAPC dressings increases blood vessel formation in normal and diabetic wounds

**FIGURE 4:**
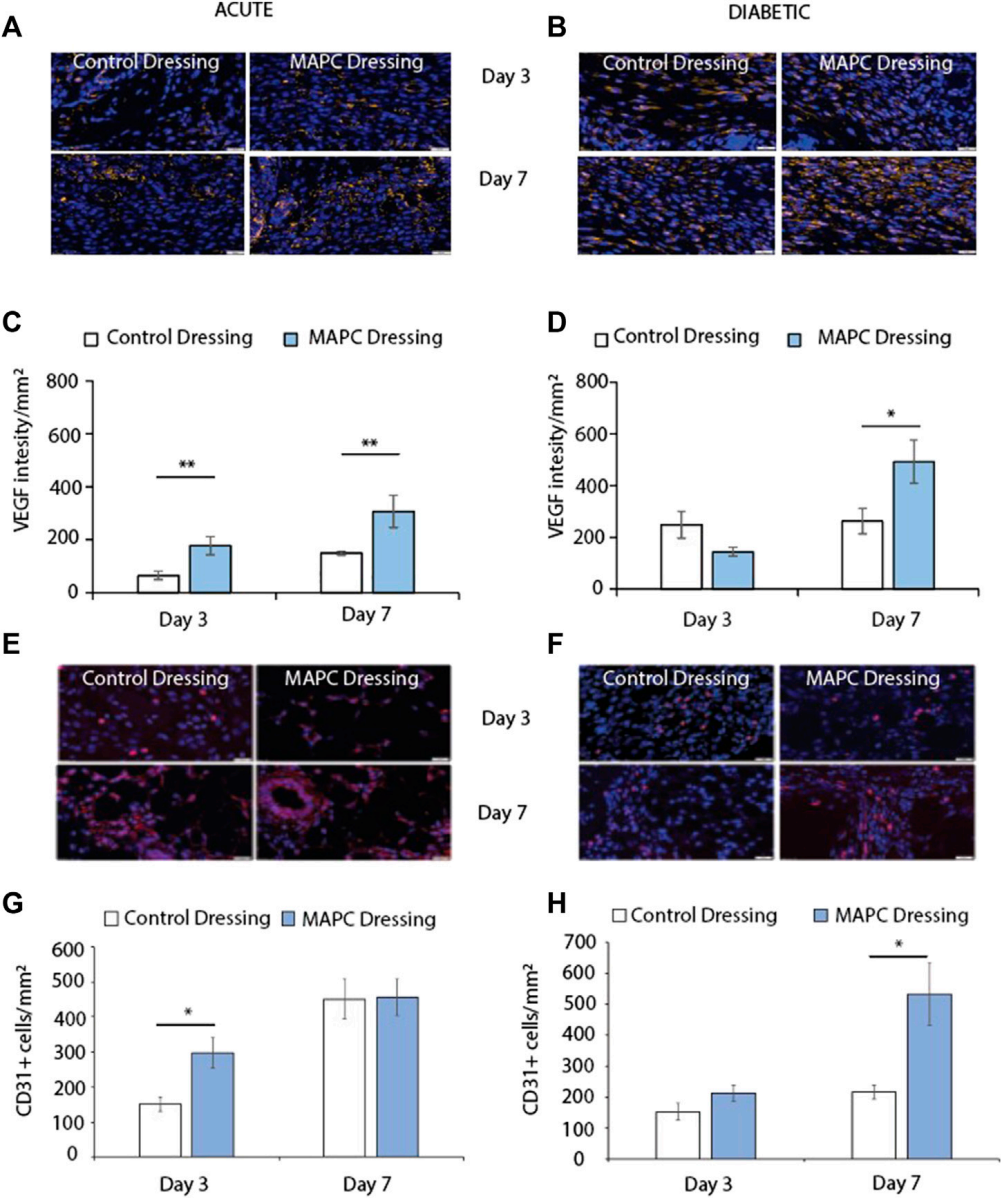
Representative images of VEGF staining in normal **(A)** and diabetic **(B)** wounds at day 3 and day 7 treated with MAPCs dressings or control dressings. Graphical representation of the intensity per mm^2^of the control and treated wounds **(C,D)**. Representative images of the immunohistochemical staining of CD31 positive cells in day 3 and day 7 normal **(E)** and diabetic wounds **(F)** and their cell counts per mm^2^ **(G,H)**. Markers of significance are * = *p* ≤ 0.05 and ** = *p* ≤ 0.01 and scale bars are 50 µm. Significance was determined using a two tailed student t test.

Angiogenesis is key to successful wound healing as without blood vessels the wound tissue and the cells within that tissue are starved of oxygen. This results in hypoxia and delays wound healing (Velazquez, 2007). Two markers of angiogenesis are VEGF and the endothelial cell marker CD31. Treatment of the excisional wounds in normal mice with MAPC dressings showed increased VEGF expression at day 3 (*p* = 0.005) and day 7 (*p* = 0.05) when compared to wounds treated with control dressings (Figures 4A, C). No significant difference was observed in VEGF expression in day 3 diabetic wounds, but increased levels of VEGF expression was observed in the wounds treated with the MAPC dressings at day 7 (*p* = 0.036) when compared to control wounds (Figures 4B, D). In the acute wounds there were significantly more CD31 positive cells in the MAPC treated wounds (*p* = 0.02) when compared to the control wounds (Figures 4E, G), which also reflected the similar expression levels of VEGF in these wounds. There were no significant differences in CD31 expression observed in the acute wound at day 7. In the diabetic wounds there was only a significant difference in CD31 positive cell numbers at day 7 (*p* = 0.03), with more CD31 cells in the MAPC treated wounds (Figures 4F, H). This also reflects the VEGF data which also showed significantly more expression in the MAPC diabetic wounds at day 7.

### MAPC dressings had no effect on collagen deposition in normal and diabetic wounds

**FIGURE 5:**
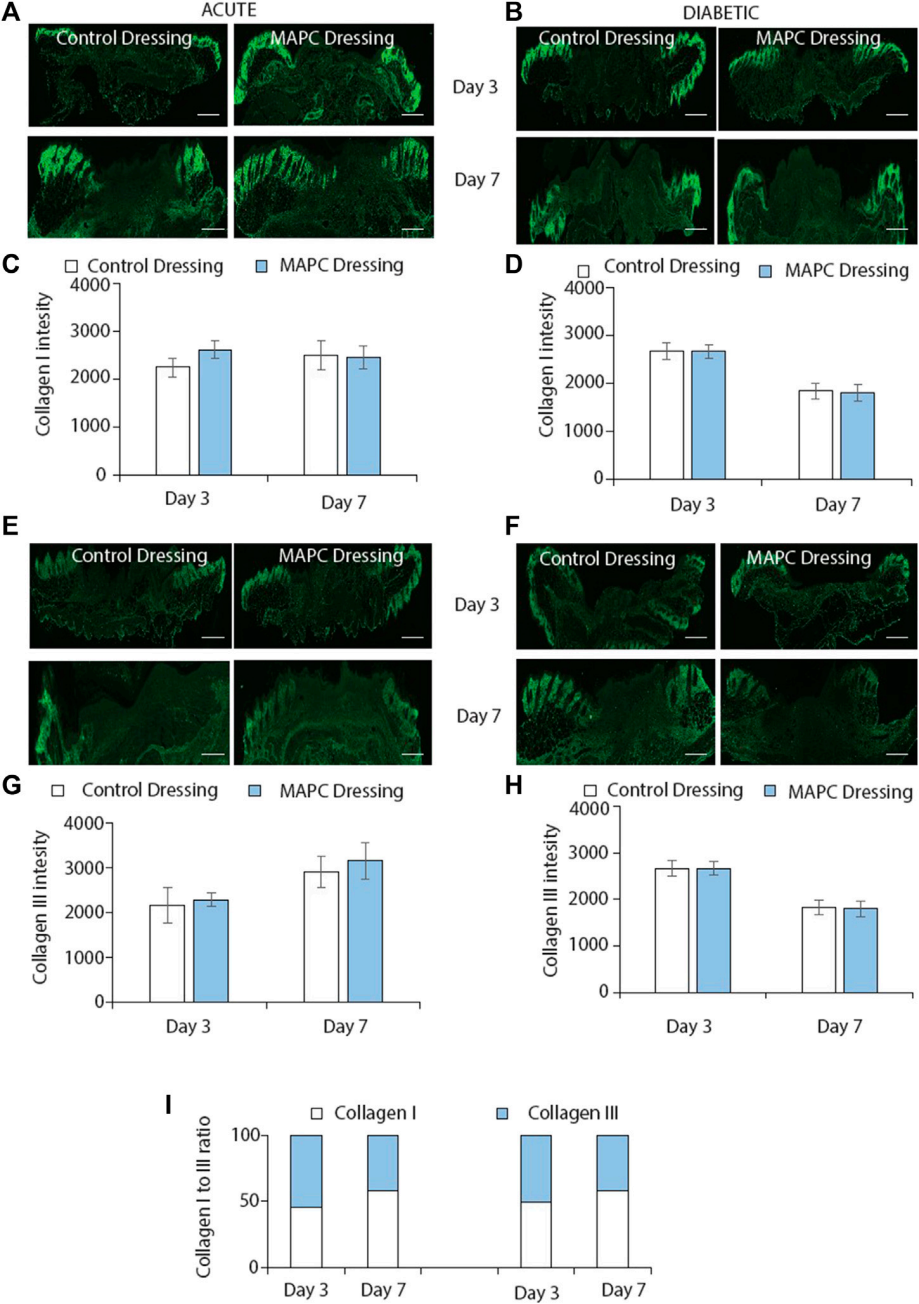
Immunofluorescent staining of collagen I in normal **(A)** and diabetic **(B)** wounds at day 3 and day 7 treated with MAPC dressings and control dressings and the graphical representation of the intensity measurements of those wounds **(C,D)**. Collagen III staining of normal **(E)** and diabetic **(F)** wounds at day 3 and day 7 when treated with the MAPC dressings and control dressings and their intensity measurements **(G,H)**. Ratio of collagen I and collagen III staining in normal and diabetic wounds at day 3 and day 7 for MAPC dressing and control dressing treated wounds **(I)**. Scale bars = 500 µm. Significance was determined using a two tailed student t test. *de*.

Collagen deposition is another key element of the wound healing process. Collagen is initially deposited in the form of collagen III, which is then replaced by collagen I (Kirby et al., 2015). In this study collagen I and III expression were investigated in both normal and diabetic wounds at day 3 and day 7 treated with the MAPC dressings. No significant differences were observed at any of the time points assessed (Figures 5A–H). The ratio of collagen I to collagen III were also considered as this is a marker of wound remodelling but again no significant differences were observed (Figure 5I).

## Discussion

In this study we have assessed the effectiveness of delivering MAPCs using a functionalized heptylamine surface on a silicone backing, using plasma polymerization. By manufacturing these MAPC dressings and applying them topically to normal and diabetic murine wounds, we have shown that we can positively influence healing and increase the rate of wound closure. This was achieved with only one application of the cells at the time of wounding, via the dressing, which remained in place for 72 h. In both the normal and diabetic wounds, treatment with the MAPC dressing increased the rate of wound closure. Previous studies have investigated the effects of MAPCs encased in alginate and discovered that when applied *in vitro* to scratch wounded corneal stromal cells, there was a significant increase in the rate of wound closure (Al-Jaibaji et al., 2018). In another study, a mixture of rodent MAPCs, epidermal stem cells and fibroblasts were injected intravenously and subcutaneously around excisional wounds of nude mice leading to accelerated wound healing rates (Ji et al., 2009). Interestingly, this study also showed that the MAPCs were able to incorporate themselves into the hair follicle bulge in the epidermis, which is a known location of stem cell populations, and were then able to contribute to wound healing in the surrounding skin (Ji et al., 2009). MAPCs have also been shown to improve the vasculature in an ischaemic limb injury model in mice where intramuscular injections of murine MAPCs showed improved muscle regeneration, increased blood flow and improved function of the effected limb. This was compared to treatments with murine MAPC vascular derived progenitors and murine bone marrow cells, which were not as effective (Aranguren et al., 2008). Vaes et al. have shown that MAPCs not only improve the invasion of vessels into a Matrigel plug injected subcutaneously in mice but that when MAPCs were injected intraperitoneally into wounded mice improvements in the vascularization and healing of the wounds was observed (Vaes et al., 2020). MAPCs can improve the vasculature around injected islet cells increasing the number of engrafted islet cells and improving the rate of diabetes reversal in mice (Cunha et al., 2017). In our study the data also showed that there was an improvement in blood vessel formation at day 3 and 7 in the model of normal healing and at day 7 in the diabetic impaired healing model determined by VEGF and CD31 staining. More recently, treatment of excisional wounds with MAPC secretome has been shown to increase the rate of healing by dampening the immune response and increasing angiogenesis (Ahangar et al., 2020b).

MAPCs have a dampening effect on the host immune system, which helps prevent rejection of the MAPCs and autoimmunity (Reading et al., 2015). They achieve this phenomenon by the suppression of T-cell proliferation in conjunction with prostaglandin E2 (PGE2) production. These characteristics make MAPCs prime candidates for allogenic products. In addition to the effects of MAPCs on the regrowth of the vasculature, described by us and others, we have now shown that MAPCs also influence the inflammatory response *in vivo*. Previous studies looking into the effects of MAPCs on the immune response have been *in vitro* and focused on the effect on T-cells to assess their potential as an allogenic product (Burrows et al., 2013; Reading et al., 2013; Reading et al., 2015). These studies show that MAPCs can inhibit the IL-7 dependent T-cell expansion to prevent autoimmunity and graft rejection. Interestingly, within these studies MAPCs were also shown to inhibit the expression of TNF and IFNγ. Whilst this was only confirmed *in vitro* it does provide an explanation for the effect of MAPCs on neutrophil and macrophage infiltration observed in this *in vivo* study. TNF and IFNγ are two cytokines that are known to increase the recruitment of both neutrophils and macrophages into the wounds site (Zhang, 2007; Ashcroft et al., 2012). With this study we also observed an inhibition of the inflammatory process with a reduction of neutrophil and macrophage numbers seen in the MAPC dressing treated wounds. Prostaglandin E2 (PGE2), which is upregulated by MAPC treatment, has also shown to have some links to the resolution of the inflammatory response (Walker et al., 2012), which further supports the reduction in the inflammatory response seen in this study. Finally, the data showed that there was little effect on collagen deposition within the wound. As accelerated healing is often associated increased scarring, treatment with the MAPC dressings was shown to not only accelerate the rate of healing but that this improvement was not achieved with a poorer cosmetic outcome.

In summary, we have shown that treatment of wounds with the MAPC dressings can accelerate healing in both normal and diabetic mice when compared to cell-free dressings. The MAPC dressings also significantly accelerated healing when compared to MAPC injections into the wound margins. This suggested that delivery of the MAPCs, via the dressing, to the entire wound surface rather than relying on the cells to migrate into the wound site can result in an overall improved rate of healing. The mechanisms by which MAPCs increase the rate of healing was achieved through a dampening of the immune response and an increase in angiogenesis. Overall, this allogenic dressing has the potential to improve the healing of chronic diabetic wounds in patients to help alleviate their suffering and the financial costs to the health services worldwide.

## Data Availability

The datasets presented in this article are not readily available because there are IP restrictions in place for this product and as such the data can not be made available at this time. Requests to access the datasets should be directed to stuart.mills@unisa.edu.au.
